# Role of GD3-CLIPR-59 Association in Lymphoblastoid T Cell Apoptosis Triggered by CD95/Fas

**DOI:** 10.1371/journal.pone.0008567

**Published:** 2010-01-05

**Authors:** Maurizio Sorice, Paola Matarrese, Valeria Manganelli, Antonella Tinari, Anna Maria Giammarioli, Vincenzo Mattei, Roberta Misasi, Tina Garofalo, Walter Malorni

**Affiliations:** 1 Department of Experimental Medicine, “Sapienza” University of Rome, Rome, Italy; 2 Laboratory of Experimental Medicine and Environmental Pathology, “Sapienza” University, Rieti, Italy; 3 Department of Drug Research and Evaluation, Section of Cell Aging and Degeneration, Istituto Superiore di Sanità, Rome, Italy; 4 Department of Technology and Health, Section of Ultrastructural Infectious Pathology, Istituto Superiore di Sanità, Rome, Italy; University of Hong Kong, Hong Kong

## Abstract

We previously found that a directional movement of the raft component GD3 towards mitochondria, by its association with microtubules, was mandatory to late apoptogenic events triggered by CD95/Fas. Since CLIPR-59, CLIP-170-related protein, has recently been identified as a microtubule binding protein associated with lipid rafts, we analyzed the role of GD3-CLIPR-59 association in lymphoblastoid T cell apoptosis triggered by CD95/Fas. To test whether CLIPR-59 could play a role at the raft-microtubule junction, we performed a series of experiments by using immunoelectron microscopy, static or flow cytometry and biochemical analyses. We first assessed the presence of CLIPR-59 molecule in lymphoblastoid T cells (CEM). Then, we demonstrated that GD3-microtubule interaction occurs via CLIPR-59 and takes place at early time points after CD95/Fas ligation, preceding the association GD3-tubulin. GD3-CLIPR-59 association was demonstrated by fluorescence resonance energy transfer (FRET) analysis. The key role of CLIPR-59 in this dynamic process was clarified by the observation that silencing CLIPR-59 by siRNA affected the kinetics of GD3-tubulin association, spreading of GD3 towards mitochondria and apoptosis execution. We find that CLIPR-59 may act as a typical chaperone, allowing a prompt interaction between tubulin and the raft component GD3 during cell apoptosis triggered by CD95/Fas. On the basis of the suggested role of lipid rafts in conveying pro-apoptotic signals these results disclose new perspectives in the understanding of the mechanisms by which raft-mediated pro-apoptotic signals can directionally reach their target, i.e. the mitochondria, and trigger apoptosis execution.

## Introduction

Cytoplasmic linker proteins (CLIPs), microtubule-binding proteins, are involved in intracellular organization and organelle movement [1]. In particular, several CLIP-170-related proteins, characterized by the presence of a cytoskeleton-associated protein-Gly motif that interacts with tubulin, are active at the organelle-microtubule interface [2]. Recently, CLIPR-59, a new CLIP-170-related protein, has been identified [3], which is involved in the regulation of microtubule dynamics. In addition to its microtubule binding, CLIPR-59 can also be associated with glycosphingolipid enriched microdomains on cell plasma membrane, i.e. with the so-called lipid rafts [4]. It has been proposed that this raft-associated CLIP could play a role at the raft-microtubule junction [4] and in the regulation of membrane trafficking [3]. Moreover, recent evidence showed that CLIPR-59 functions as a scaffold protein that interacts with phospho-Akt and regulates Akt cellular compartmentalization [5]. The role of CLIPR-59 in the regulation of signal transduction pathway(s) is related to its association with lipid rafts on the cell surface. Indeed, the last 30 amino acids of CLIPR-59 are required to target it to the plasma membrane and a double palmitoylation on tandem cysteines within this domain is responsible for the raft targeting.

Lipid rafts have been associated with several cell functions [6], [7], including cell death. It has in fact been suggested that lipid rafts could play a key role in receptor-mediated apoptosis of T cells [8], [9]. This is apparently due to two events that follow the receptor engagement: i) the recruitment of CD95/Fas [9]–[11] as well as other Tumor Necrosis Factor-family receptors [12] to plasma membrane lipid rafts, and ii) the recruitment of specific proapoptotic bcl-2 family proteins to mitochondrial “raft-like microdomains” [13]. Indeed, small lipid domains are also present on mitochondrial membrane, where they may contribute to apoptosis-associated modifications of the organelle, i.e. its remodeling and fission, as well as to the release of apoptogenic factors and apoptosis execution [10], [13]. These raft-like microdomains are enriched in gangliosides (GD3, GM3) and cardiolipin [14], but show a relatively low content of cholesterol; some molecules, including the voltage-dependent anion channel-1 and the fission protein hFis1, are enriched, whereas Bcl-2 family proteins (truncated Bid and Bax) are recruited, following CD95/Fas triggering [13]. Both mitochondria depolarization and cytochrome c release are dependent on raft-like microdomain integrity, since the disruption of raft-like microdomains by methyl-β-cyclodextrin prevented mitochondria depolarization or cytochrome c release induced by GD3 or by the active form of Bid (t-Bid) [13]. We recently identified microtubular network as pivotal in the intracellular directional redistribution of lipid raft components [15]. We showed the association of GD3 with alpha and beta tubulin. In particular, in silico docking analysis showed that GD3 has a high affinity for the pore formed by four tubulin heterodimers (type I pore), thus suggesting a possible interaction between tubulin and GD3. Hence, microtubules could act as tracks for ganglioside redistribution following apoptotic stimulation, possibly contributing to the mitochondrial alterations leading to cell death.

The present study was thus undertaken to ascertain whether the movement of GD3 from the plasma membrane towards the mitochondrion *via* microtubules could be instructed by its association with CLIPR-59. In fact, we found that this small molecule seems to behave as a typical chaperone allowing a prompt interaction between tubulin and ganglioside GD3, here considered as a paradigmatic microdomain component [10] regulating CD95/Fas-triggered apoptosis in lymphoblastoid T cells (CEM).

## Results

### Analysis of CLIPR-59 distribution in CEM cells

**Figure 1:**
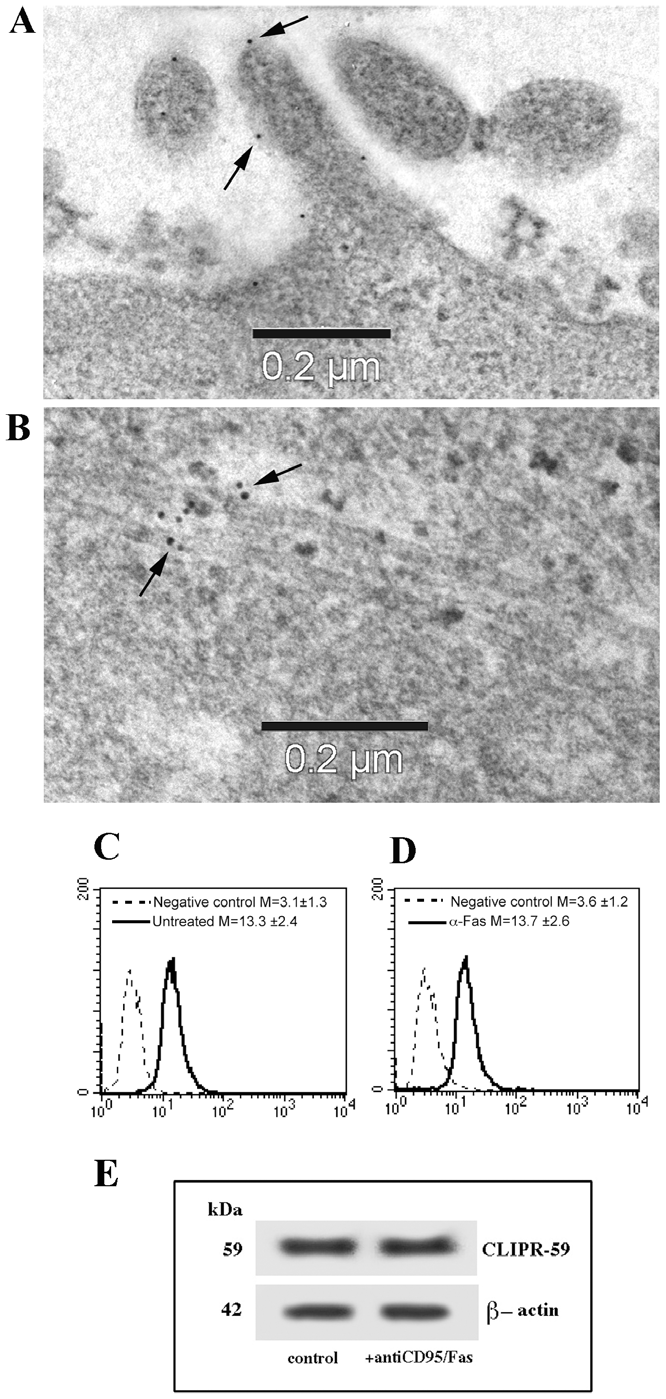
GD3 associates with CLIPR-59 after anti-CD95/Fas apoptotic triggering. Immunoelectron microscopy analysis of CLIPR-59 distribution by immunogold staining using post-embedding technique clearly indicated a surface staining in control CEM cells (A) and a microtubule associated gold labeling in anti-CD95/Fas treated cells (B). Arrows indicate gold particles. Note the absence of non-specific labeling. (C) Flow cytometric analysis of CLIPR-59 expression in control CEM cells; (D) Flow cytometric analysis of CLIPR-59 expression in anti-CD95/Fas treated cells. (E) Western Blot analysis of CLIPR-59 expression in control or in anti-CD95/Fas treated cells. As a loading control β-actin is shown.

CLIPR-59, a CLIP-170-related protein, has recently been identified as a microtubule binding protein associated with lipid rafts [4]. To test whether raft-associated CLIPR-59 could play a role at the raft-microtubule junction, we performed a series of experiments by using immunoelectron microscopy, static and flow cytometry as well as biochemical analyses. We first assessed the presence of CLIPR-59 molecule in CEM cells. Studies on CLIPR-59 association to lipid rafts and microtubules were indeed previously conducted in CLIPR-59-transfected cells only [3], [4]. In CEM cells we found a small, but well-defined, presence of CLIPR-59 gold labeling at the cell surface, (**Fig. 1A**, note the absence of any sign of non-specific labeling). Interestingly, after CD95/Fas triggering, CLIPR-59 was detectable on microtubules (**Fig. 1B**, arrows indicate 10 nm gold particles). Positivity for CLIPR-59 was also confirmed by flow cytometry analyses (**Fig. 1C–D**). Western blot analysis of CLIPR-59, either in the presence or in the absence of CD95 stimulation, revealed that CLIPR-59 expression was substantially unchanged, supporting the view that apoptosis triggering does not change CLIPR-59 expression, but affects its redistribution from plasma membrane domains to microtubules (**Fig. 1E**).

### Analysis of CLIPR-59 association with GD3

**Figure 2:**
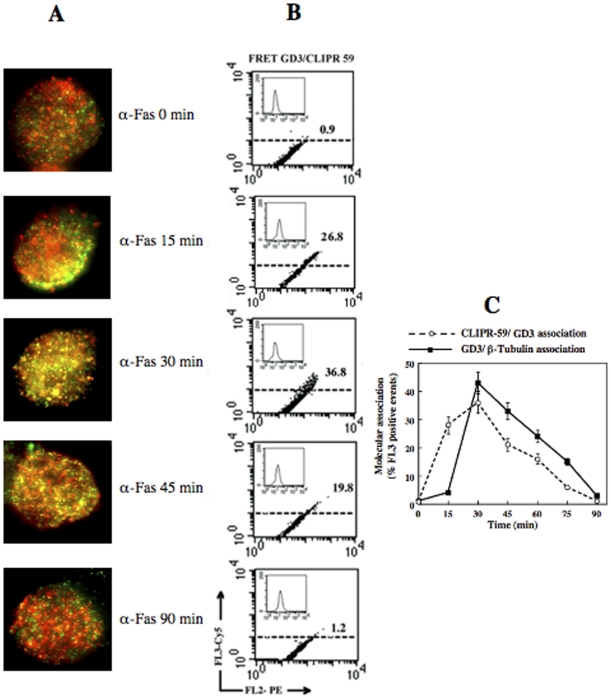
Static and flow cytometric analyses of GD3/CLIPR-59 association. (A) Immunofluorescence analysis after double staining of GD3 (red) and CLIPR-59 (green) in untreated CEM cells and after treatment with anti-CD95/Fas (only merge pictures are shown). Note GD3/CLIPR-59 co-localization (yellow staining) at different time points (starting from 15 min) and the absence of any co-localization 90 min after anti-CD95/Fas treatment (bottom panel). (B) Quantitative evaluation of GD3/CLIPR-59 association by FRET technique, as revealed by flow cytometry analysis. This association was negligible in untreated CEM cells, started after 15 min after anti-CD95/Fas administration, reached its peak 30 min later and dropped down 90 min after treatment. Numbers represent the FRET efficiency indicating the GD3/CLIPR-59 association. Results obtained in one experiment representative of four are shown. Inset: flow cytometry analysis of CLIPR-59 expression at different times of anti-CD95/Fas treatment. (C) Time course analysis of the association GD3/CLIPR-59 compared with GD3/β-Tubulin association in CEM cells after anti-CD95/Fas administration. Note the different trend of the two curves, i.e. the earlier association of GD3/CLIPR-59 with respect to GD3/β-Tubulin association.

**Figure 3:**
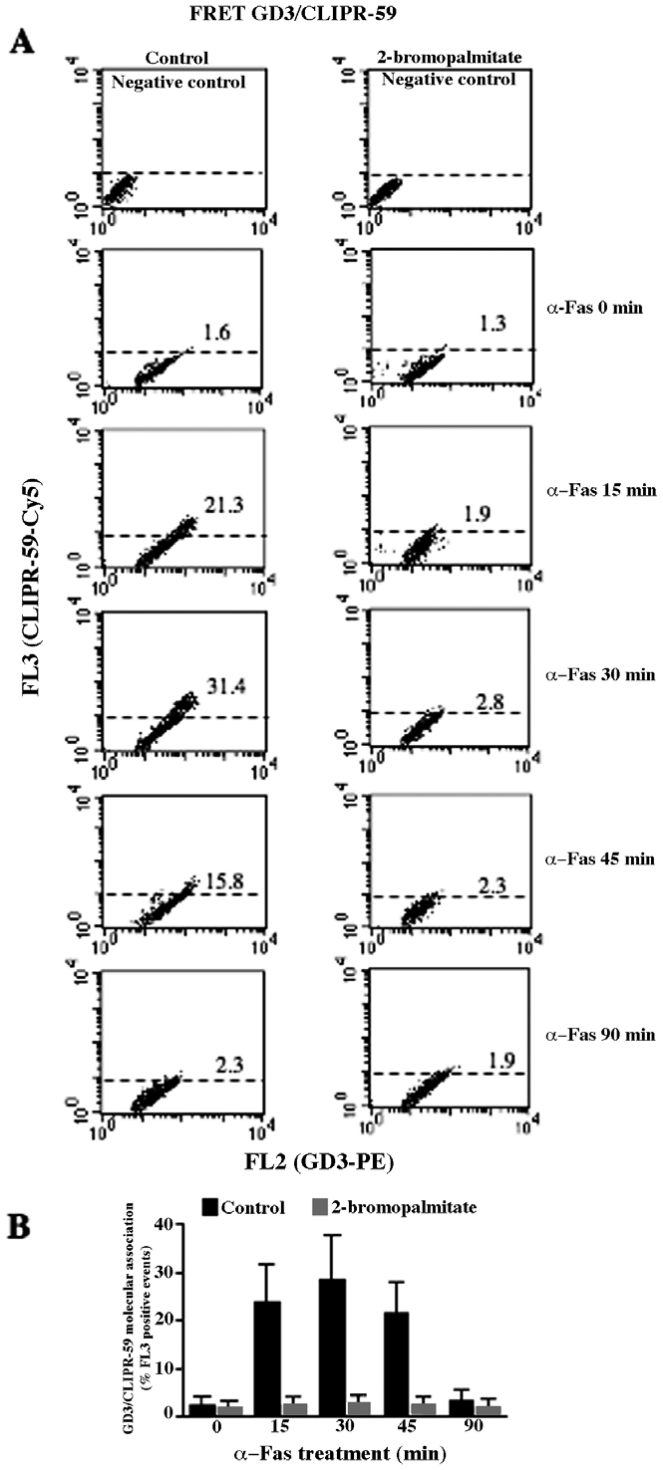
Effect of 2-Bromopalmitate on GD3/CLIPR-59 association. Quantitative evaluation of GD3/CLIPR-59 association by FRET technique, as revealed by flow cytometry analysis. This association, negligible in untreated cells, started 15 min after the administration of anti-CD95/Fas (250 ng/ml for different incubation times at 37°C) reached its peak 30 min later and dropped down 90 min after treatment (left column). In cell pre-treated with 100 µM 2-Bromopalmitate for 3 h at 37°C to inhibit palmitoylation, GD3/CLIP-59 association was inhibited at any time (right column). Numbers represent the percentage of FL3 positive cells indicating the GD3/CLIPR-59 association. Results obtained in one experiment representative of three are shown. (B) Time course analysis of the association GD3/CLIPR-59 obtained pooling together results of three independent experiments. Data are reported as mean values±SD.

To assess the possible implications of the association CLIPR-59/GD3 for GD3 trafficking, specific experiments were then carried out. Static cytometry analyses performed at different time points indicated that a co-localization of GD3 with CLIPR-59 was detectable 15 minutes after Fas-triggering (**Fig. 2A**). Parallel FRET analyses indicated that GD3/CLIPR-59 association was evident 15 minutes after anti-CD95/Fas administration, reaching a peak after 30 minutes (**Fig. 2B**, representative experiment). Of note, CD95/Fas treatment virtually did not modify the expression of CLIPR-59 (Fig. 2B, insets). However, the results obtained by FRET analyses also underline that the association CLIPR-59/GD3 occurred earlier than the association GD3/β-Tubulin, as shown by the slight shift of the two association curves based on values obtained by FRET analyses (**Fig. 2C** and **Fig. S1**). In addition, to test the role of cysteine palmitoylation of CLIPR59 in the GD3 traffic, parallel experiments were performed by pretreating cells with 100 µM 2-Bromopalmitate to inhibit palmitoylation. The analysis revealed that pretreatment significantly prevented GD3/CLIPR59 interaction, as revealed by FRET analysis (Fig. 3A and B).

### Effect of CLIPR-59 siRNA on GD3/β-Tubulin association and apoptosis

**Figure 4:**
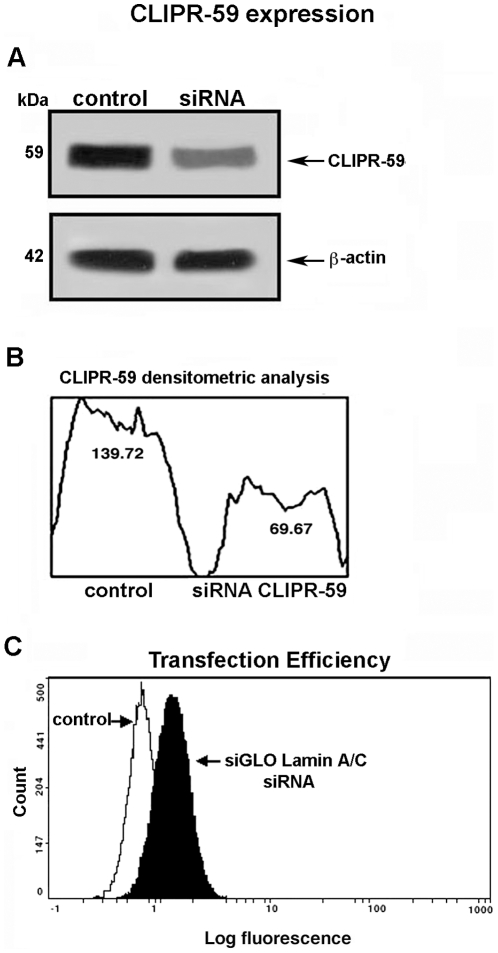
Analysis of CLIPR-59 expression and transfection efficiency. A) CEM cells, transfected or not with 100 nM smart pool siRNA targeting CLIPR-59, were lysed, resolved by SDS-PAGE and transferred to nitrocellulose. Samples were probed with anti-CLIPR-59 polyclonal antibodies or with anti-β-actin as a control. The immunoreactivity was assessed by chemiluminescence reaction using the ECL western blocking detection system. B) Densitometric scanning analysis of the CLIPR-59 expression was performed by MAC OS 9.0, using NIH image 1.62 software. C) CEM cells, transfected or not with 100 nM siGLO laminin A/C siRNA, were fixed with 2% paraformaldehyde and the fluorescence was detected by flow cytometry. Transfection efficiency was about 60%.

In order to demonstrate the role of CLIPR-59 as a regulator of GD3 trafficking, a small interfering RNA (siRNA) was employed to ablate CLIPR-59 and its function. Western blot analyses of siRNA-treated cells revealed that CLIPR-59 expression appeared to be significantly reduced as compared to control cells (**Fig. 4A**), as detected by densitometric analysis (**Fig. 4B**). Optimum transfection efficiency, confirmed by positive control siGLO laminin A/C siRNA with fluorescent label, was about 60% (**Fig. 4C**).

**Figure 5:**
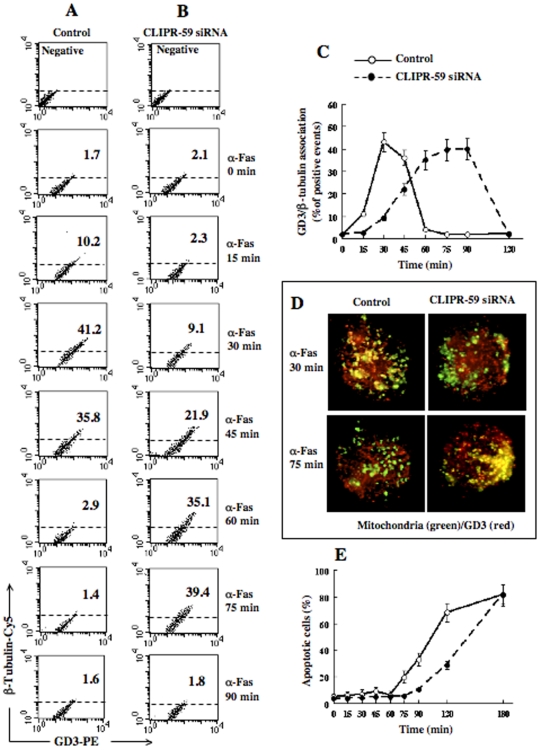
Effect of CLIPR-59 siRNA on GD3/β-Tubulin association and apoptosis. (A–B) Time-course cytometric analysis of GD3/β-Tubulin association by FRET technique in control non-silenced cells (A) and in CLIPR-59 siRNA treated cells (B). Numbers represent the percentage of cells in which GD3/β-Tubulin association occurred. Results obtained in one experiment representative of three are shown. Note that FRET from acceptor to donor (indicating molecular association): i) reached its peak 30 min after anti-CD95/Fas administration in control cells and 75 min after CD95/Fas triggering in CLIPR-59 silenced cells and ii) dropped down 60 min after anti-CD95/Fas treatment in control cells and 90 min in CLIPR-59 siRNA treated cells. (C) Comparative time course analysis of GD3/β−Τ ubulin association (by FRET analysis) in control and CLIPR-59 siRNA treated cells. (D) Immunofluorescence analysis after double staining of GD3 (red) and mitochondria (green) in control and CLIPR-59 siRNA treated cells 30 and 75 min after treatment with anti-CD95/Fas (only merge pictures are shown). Note that GD3/mitochondria co-localization (yellow staining) was detectable at different time points after CD95/Fas treatment. (E) Comparative time course analysis of apoptosis (by evaluating the hypodiploid peak). Results are reported as mean values from three independent experiments±SD. Note the “delay” of both apoptosis and GD3/β-tubulin association induced by CLIPR-59 silencing by siRNA.

The analysis of GD3/β-tubulin association was thus carried out by FRET technique at different time points (15–90 minutes) after CD95/Fas administration. The results obtained in a representative experiment are reported in **Figure 5A and B**. The data show that silencing of CLIPR-59 by siRNA affected the kinetics of GD3-β-tubulin association, after CD95/Fas administration. Indeed, the maximum FRET efficiency (FE) was detected 30 minutes after anti-CD95/Fas triggering in control non-silenced cells (Fig. 5**A**) (FE:1.3211 *vs* 0.2385 in CLIPR-59 siRNA treated cells), whereas in CLIPR-59 siRNA treated cells similar FRET efficiency values (FE: 1.1094) were detected only after 75 minutes (Fig. 5**B**). Notably, at this time point, GD3/β-tubulin association was negligible in control samples (FE: 0.2160). This delay was better appreciable by pooling together the results obtained from three different experiments (**Fig. 5C**, where the GD3/β-tubulin association is reported as a function of time). In fact, ANOVA two-way for repeated measures showed a not significant effect of treatment (F 1,48 = 3.60, P = 0.0942), but a significant time (F 6,48 = 188.89, P<0.0001) and treatment x time interaction (F 6,48 = 225.53; P<0.0001). This indicates that the two curves have a similar trend but also that a significant “delay” of GD3/β-Tubulin association is induced by CLIPR-59 siRNA treatment (**see Fig. S2A–C**). Similarly, immunofluorescence analysis after double staining of GD3 (red) and mitochondria (green) in control and CLIPR-59 siRNA treated cells revealed different kinetics of GD3/mitochondria association, after treatment with anti-CD95/Fas. Indeed, GD3/mitochondria colocalization was detected 30 minutes after anti-CD95/Fas triggering in control cells, but was impaired in CLIPR-59 siRNA treated cells (**Fig. 5D**). As a control, parallel staining was performed with R24 anti-GD3 monoclonal antibody (MoAb), which revealed a similar distribution pattern (Fig. S3). Further analyses were also conducted to evaluate apoptotic rates at different time points up to 180 min. The analysis of the hypodiploid peak revealed a significant delay of CD95/Fas triggered apoptosis in siRNA treated cells as compared to non-silenced cells (**Fig. 5E**).

## Discussion

The present work deals with the trafficking of glycosphingolipid GD3 to the mitochondrion upon pro-apoptotic triggering induced by CD95/Fas ligation and identifies the pivotal role of microtubule-associated protein CLIPR-59 in instructing and regulating GD3-microtubule association.

The re-distribution of GD3 in lymphoblastoid T cells may play a decisive role in the apoptosis cascade. Previous works, including ours, identified the mitochondria as possible targets for GD3 and hypothesized that the rearrangement of GD3 may be involved in the mitochondrial remodeling leading to apoptosis execution phase [13], [16], [17]. It was in fact suggested that mitochondria remodeling in terms of structural modifications, i.e. their curvature changes, as well as their fission process, could be under the influence of several molecules, including lipid microdomains. It was also proposed that it could play a key role in late apoptotic mitochondria-mediated events, i.e. the release form this organelle of apoptogenic factors such as cytochrome c [13], [18], [19]. In this scenario we hypothesized that lipid rafts constituents (GD3), normally localized mainly at the cell surface [20] and able to engulf a series of molecules of importance in the cell suicide process [9], [11], [12], can proceed from the cell plasma membrane (and/or from *trans* Golgi network) to the mitochondria *via* a microtubule-dependent mechanism. Microtubules may be used as tracks to direct intracytoplasmic transport of lipid raft glycosphingolipid(s) to mitochondria. This was demonstrated by the observed association of GD3 with tubulin and by the experiments previously carried out by inhibiting microtubule polymerization [15]. Under these experimental conditions, the trafficking of GD3 molecule towards mitochondria appeared to be impaired. However, the fact that the integrity of microtubules is mandatory for GD3 association to tubulin is still puzzling. This question remains to be elucidated and some insight may come from the studies carried out in this paper in which we analyzed the microtubule associated protein CLIPR-59. In fact, CLIPR-59, in addition to its microtubule binding, has recently been shown to be associated with lipid rafts by a double palmitoylation on tandem cysteines within the C-terminal domain [4].

Since CLIPR-59 is associated not only with the plasma membrane, but is also targeted to *trans* Golgi network membranes, it may regulate both plasma membrane and *trans* Golgi network interactions via microtubules. Here, in addition, we demonstrated, by FRET, that CLIPR-59 is also capable of directly interacting with lipid raft-associated GD3. Interestingly, it was proposed that CLIPR-59 binds microtubules only when already localized to its membrane target [3]. It can therefore be hypothesized that it can play a role either as cytoplasmic linker between lipid rafts and microtubules or to locally destabilize the assembly of microtubules close to lipid rafts [4]. More in general, according to CLIP model [1], CLIPR-59 would establish an interaction between cell membranes and microtubules, thus regulating membrane dynamics. In particular, CLIPR-59 may facilitate rafts/microtubules interaction following anti-CD95/Fas treatment. This model would explain the shift observed between association kinetics of GD3 to CLIPR-59 and β-Tubulin, as revealed by FRET analyses. Our results support the view that CLIPR-59 is involved in intracellular trafficking, acting as a chaperone molecule allowing a fast and prompt interaction between GD3 and tubulin, once apoptosis has been triggered by CD95/Fas. In particular, findings of the experiments with 2-Bromopalmitate suggest that palmitoylation of CLIPR59 plays a key role in the overall process of GD3/tubulin interaction. Moreover, the key role of CLIPR-59 in this dynamic process is clarified by the observation that silencing CLIPR-59 by siRNA resulted in a delayed GD3-β-tubulin association and, consequently, a delayed apoptosis execution, probably *via* an inhibited spreading of GD3 towards mitochondria. However, we cannot exclude the possibility that other, still unidentified, molecules may drive GD3 traffic. In particular, we demonstrated that ezrin, a cytoskeletal protein, may directly interact with GD3 in uropods of lymphoblastoid CEM cells during cell apoptosis triggered by CD95/Fas [21]. Furthermore, on the basis of literature [22] and according to [5], we can hypothesize that the interaction of CLIPR-59 with Akt could play a role in the cascade of events leading to the observed significant delay of apoptotic execution. In fact, since Akt activation is known to inhibit apoptosis, we cannot rule out the possibility that affecting CLIPR59 function could impair signaling through lipid rafts, which results in Akt inactivation and cell death.

Taken together, our findings bolster the role played by lipid rafts in the apoptotic program and their role in the preparatory homework for cell suicide apoptosis introducing a new actor in the process: the CLIPR-59 microtubule binding protein and its chaperone activity.

## Materials and Methods

### Cells and treatments

CEM cells obtained from a human acute lymphoblastic leukemia were cultured as described [23]. Apoptosis was induced by incubating cells at a concentration of 5×10^5^ per ml in complete medium by adding anti-Fas (CD95) IgM MoAb (clone CH11, Upstate Biotechnology, Lake Placid, NY USA) at 250 ng/ml for different incubation times.

### Detection of CLIPR-59 by transmission electron microscopy

CEM cells, untreated or treated with anti-Fas (30 or 60 min), were fixed in 2.5% cacodylate-buffered (0.2 M, pH 7.2) glutaraldehyde for 20 min at RT and post-fixed in 1% OsO_4_ in cacodylate buffer for 1 h at RT. Fixed specimens were dehydrated through a graded series of ethanol solutions and embedded in Agar 100 (Agar Aids, Cambridge, U.K.). Serial ultrathin sections were collected on 200-mesh grids. Thin sections were then treated with phosphate buffered saline (PBS) containing 1% (w/v) gelatin, 1% Bovine Serum Albumin, 5% Fetal Calf Serum and 0.05% Tween 20 and then incubated with anti-CLIPR-59 polyclonal Ab (kindly provided by Dr Franck Perez), overnight at 4°C. After washing for 1 h at RT, sections were labeled with anti-rabbit IgG-10 nm gold conjugate (1∶10) for 1 h at RT and washed again. Negative controls were incubated with the gold conjugate alone and then counterstained with uranyl acetate and lead citrate. Sections were observed with a Philips 208 electron microscope at 80 kV.

### Morphometric analyses

Morphometric analyses were carried out by evaluating at least 200 cells at high magnification in order to detect gold particles (20,000×) by transmission electron microscopy. Static cytometry morphometry in double labeling experiments was carried out by evaluating at least 200 cells at the same magnification (630×).

### Analysis of CLIPR-59 expression in CEM cells by Western Blot

CEM cells, untreated or treated with anti-CD95/Fas (250 ng/ml for 30 min at 37°C) were lysed in lysis buffer (10 nM Tris-HCL (pH 8.0), 150 mM NaCl, 1% Nonidet P-40, 1 mM PMSF, 10 mg/ml leupeptin). Total cell lysate from control and CD95/Fas-treated cells were analyzed by Western blot. Samples were probed with anti-CLIPR-59 polyclonal antibodies or with anti-β-actin MoAb (Sigma Chemical Co, St. Louis, MO, USA) as a control.

### Immunofluorescence by intensified video microscopy

Control and treated cells (15, 30, 45 60, 75 min and 2 h) were fixed with 4% paraformaldehyde in PBS for 30 min at room temperature and then permeabilized with 0.5% Triton X-100 in PBS for 5 min at room temperature, as previously reported [24]. After three washes in PBS, samples were incubated with GMR19 anti-GD3 MoAb (Seikagaku Corporation, Chuo-ku, Tokyo, Japan) [25] or, alternatively, with R24 anti-GD3 (Matreya Inc., Pleasant Gap, PA) for 1 h at 4°C, followed by three washes in PBS and addition (30 min at 4°C) of Alexa Fluor 594-conjugated anti-mouse IgM or IgG (Molecular Probes, Leiden, The Netherlands). The GMR19 anti-GD3 antibody is highly specific, as demonstrated by thin layer chromatography immunostaining and immunofluorescence analysis [25]. We further verified the specificity of the antibody in CEM cells [10], [13], [14]. After washes, cells were incubated with the anti-CLIPR-59 polyclonal Ab for 1 h at 4°C, followed by addition (30 min at 4°C) of Alexa Fluor 488-conjugated anti-rabbit IgG (Molecular Probes). Cells were finally washed in PBS, and resuspended in *0.1 M* Tris-HCl, pH 9.2, containing 60% glycerol (v∶v). and observed with a Nikon Microphot fluorescence microscope. Images were captured by a color chilled 3CCD camera (Hamamatsu, Japan) and analyzed by the OPTILAB (Graftek, France) software.

### Fluorescence resonance energy transfer by flow cytometry

We applied FRET analysis by flow cytometry in order to study the co-localization [26] of GD3/CLIPR-59 or GD3/β-Tubulin. Briefly, cells, untreated or treated with anti-CD95/Fas (250 ng/ml for different incubation times at 37°C) were fixed and permeabilized as reported above [22]. In parallel experiments, cells were pretreated with 100 µM 2-Bromopalmitate (Sigma Chem Co) [27] for 3 h at 37°C to inhibit palmitoylation. After two washing in cold PBS the cells were labeled with Abs tagged with donor (PE) or acceptor (Cy5) dyes. GD3 staining was performed using unlabelled mouse antibody (Seikagaku Corporation) and saturating amount of PE-labeled anti-mouse IgM (Sigma). CLIPR-59 was revealed by the anti-CLIPR-59 polyclonal Ab; tubulin was detected by anti-β-Tubulin antibody (Abcam Ltd., Cambridge, UK), followed by biotinylated anti-rabbit IgG and then saturating concentrations of streptavidin-Cy5 (both from BD Pharmingen).

### SiRNA CLIPR-59

CEM cells were cultured in a serum and antibiotic free medium and transfected with Dharma FECT 4 reagent (Dharmacon, Lafayette, CO), according to the manufacturer's instructions, using 100 nM Smart pool siRNA CLIPR-59. The transfection efficiency was confirmed by using a Dharmacon's positive silencing control, siGLO laminin A/C siRNA.

After 72 h, the culture medium was replaced with fresh medium and transfected again, as above, with 100 nM Smart pool siRNA CLIPR-59. After further 48 h, the effect of transfection was verified by Western blot and flow cytometry analyses with the CLIPR-59 polyclonal Ab.

### Preparation and labeling of isolated mitochondria

Control and CD95/Fas-treated cells were resuspended in Homo-buffer (10 mM Hepes, pH 7.4; 1 mM ethylene glycol-bis(-aminoethyl ether) N,N′,N″-tetraacetic acid (EGTA), 0.1 M sucrose, 5% BSA, 1 mM phenylmethylsulfonyl fluoride (PMSF) and complete protease inhibitor cocktail (Roche, Indianapolis, IN, USA) for 10 min on ice. Cells were homogenized with a Teflon homogenizer with B-type pestle [28] for 10 min at 4°C to remove intact cells and nuclei. The supernatants were further centrifuged at 10.000×g at 4°C for 10 min to precipitate the heavy membrane fractions (enriched in mitochondria). These fractions were then purified by standard differential centrifugation. The mitochondrial pellet obtained was fixed in paraformaldehyde for 1 h at 4°C and then washed twice with PBS/0.5% BSA. Samples were divided into equal parts, with only one part of each sample (positive samples) incubated first with a saturating amount of anti-GD3 MoAb (Seikagaku Corporation), followed by appropriate secondary Ab, as reported above for the entire cells.

### Cell-death assays

Quantification of apoptosis was performed by evaluating DNA fragmentation in ethanol-fixed cells using propidium iodide (PI, Sigma). Alternatively, apoptosis was also quantified by flow cytometry after double staining using FITC-conjugated annexin V/propidium iodide (PI) apoptosis detection kit (Eppendorf, Milan, Italy), which allows discrimination between early apoptotic, late apoptotic and necrotic cell.

### Data analysis and statistics

For morphometric analyses statistical analyses were performed by using student *t* test (Statview software for Macintosh computer). For flow cytometry studies all samples were analyzed with a FACScan cytometer (BD Biosciences, Heidelberg, Germany) equipped with a 488 argon laser. At least 20,000 events were acquired. Data were recorded and statistically analyzed by a Macintosh computer using CellQuest Software. The expression level of the analyzed proteins on intire cells or isolated mitochondria was expressed as median fluorescence and the statistical significance was calculated by using the parametric Kolmogorov-Smirnov (K/S) test. For FRET studies samples were recorded by a dual-laser FACScalibur cytometer (BD). Collected data analysis was carried out by using ANOVA two-way test for repeated samples by using Graphpad software. All data reported in this paper were verified in at least three different experiments and reported as mean±SD. Only *p* values of less than 0.01 were considered significant.

## Supporting Information

Figure S1 Time course evaluation of GD3-CLIPR-59 association after CD95/Fas triggering by FRET efficiency calculation. CEM cells, untreated or treated with anti-Fas (250 ng/ml for different incubation times) were fixed and permeabilized as reported in the text. After washings cells were pre-incubated with 10 µg/ml human IgG (Sigma) at room temperature for 10 min and then incubated with saturating concentrations of the IgM PE-conjugated and or anti biotinylated on ice for 30 min. After washing, samples were divided into equal parts and only one part (positive samples) incubated with a saturating amount of SA-Cy5 (0.6 µg/ml) for 30 min on ice. For determination of FRET efficiency, changes in fluorescence intensities of donor plus acceptor labeled cells were compared to the emission signal from cells labeled with donor-only and acceptor-only fluorophores. All data were corrected for background by subtracting the binding of the isotype controls. Efficient energy transfer resulted in an increased acceptor emission on cells stained with both donor and acceptor dyes. The FRET efficiency (FE) was calculated according to Riemann et al. (Biochem Biophys Res Commun 331: 1408–1412, 2005), where A is the acceptor and D the donor, with the formula: FE = (FL3DA−FL2DA/a−FL4DA/b)/FL3DA Where a = FL2D/FL3D and b = FL4A/FL3A.
(0.03 MB PDF)

Figure S2 Statistical analyses (ANOVA) of FRET data in Figure 4C. Data (A), tabular results (B) and narrative results (C) are included.
(1.10 MB PDF)

Figure S3 Immunofluorescence analysis of GD3/mitochondria association by R24 MoAb. Immunofluorescence analysis after double staining of GD3 (red) and mitochondria (green) in control and anti-CD95/Fas treated cells. Mitochondria were stained with MitoTracker-Green and GD3 with anti-GD3 R24 MoAb, followed by anti-mouse Alexa594.
(18.08 MB TIF)
