# Supplementary material for: Role of GD3-CLIPR-59 Association in Lymphoblastoid T Cell Apoptosis Triggered by CD95/Fas
Source: PLoS One. 2010 Jan 5;5(1):e8567. doi: 10.1371/journal.pone.0008567 (PMC2797139; doi:10.1371/journal.pone.0008567)
Supplement: Figure S1 — Time course evaluation of GD3-CLIPR-59 association after CD95/Fas triggering by FRET efficiency calculation. CEM cells, untreated or treated with anti-Fas (250 ng/ml for different incubation times) were fixed and permeabilized as reported in the text. After washings cells were pre-incubated with 10 µg/ml human IgG (Sigma) at room temperature for 10 min and then incubated with saturating concentrations of the IgM PE-conjugated and or anti biotinylated on ice for 30 min. After washing, samples were divided into equal parts and only one part (positive samples) incubated with a saturating amount of SA-Cy5 (0.6 µg/ml) for 30 min on ice. For determination of FRET efficiency, changes in fluorescence intensities of donor plus acceptor labeled cells were compared to the emission signal from cells labeled with donor-only and acceptor-only fluorophores. All data were corrected for background by subtracting the binding of the isotype controls. Efficient energy transfer resulted in an increased acceptor emission on cells stained with both donor and acceptor dyes. The FRET efficiency (FE) was calculated according to Riemann et al. (Biochem Biophys Res Commun 331: 1408–1412, 2005), where A is the acceptor and D the donor, with the formula: FE = (FL3DA−FL2DA/a−FL4DA/b)/FL3DA Where a = FL2D/FL3D and b = FL4A/FL3A. (0.03 MB PDF) [file pone.0008567.s001.pdf]

GD3/CLIPR FRET

**Untreated**	**FL-2**	**FL-3**	**FL-4**
neg PE	2	1,92	1,34
GD3 PE	14,7	2,22	1,36
neg Cy5	1,02	2,5	2,99
CLIPR Cy5	2,6	4,1	21,5
neg PE + neg Cy5	2,2	3,2	2,37
GD3 PE + CLIPR Cy5	4,3	4,5	12,9

	FL-2	FL-3	FL-4	a	b	**FRET**
GD3 PE	12,7	0,3	0,02	**42,33333**		
CLIPR Cy5	1,58	1,6	18,51		**11,56875**	
GD3 PE + CLIPR Cy5	**2,1**	**1,3**	**10,53**			**0,26168**

**anti-Fas 15 min**	**FL-2**	**FL-3**	**FL-4**
neg PE	1,17	1,13	1,45
GD3 PE	14,4	2,9	1,43
neg Cy5	1,02	2,4	4,62
CLPR Cy5	1,3	3,4	23,2
neg PE + neg Cy5	1,06	1,35	5,48
GD3 PE +CLIPR Cy5	4,2	12,2	5,9

	FL-2	FL-3	FL-4	a	b	**FRET**
GD3 PE	13,23	1,77	-0,02	**7,474576**		
tubulin Cy5	0,28	1	18,58		**18,58**	
GD3 PE + tubulin Cy5	**3,14**	**10,85**	**0,42**			**0,9592**

**anti-Fas 30 min**	**FL-2**	**FL-3**	**FL-4**
neg PE	1,5	1,27	1,35
GD3 PE	14,05	2,7	1,31
neg Cy5	1,3	6,4	4,24

CLIPR Cy5	1,06	4,74	21,42
neg PE + neg Cy5	1,21	1,63	1,75
GD3 PE + CLIPR Cy5	12,36	2,19	12,81

	FL-2	FL-3	FL-4	a	b	**FRET**
GD3 PE	12,55	1,43	-0,04	8,776224		
tubulin Cy5	-0,24	-1,66	17,18		-10,3494	
GD3 PE + tubulin Cy5	11,15	0,56	11,06			**0,63961**

**anti-Fas 45 min**	**FL-2**	**FL-3**	**FL-4**
neg PE	1,6	1,2	1,6
GD3 PE	14,9	3,2	1,5
neg Cy5	1,02	2,5	1,81
CLIPR Cy5	1,02	1,7	21,9
neg PE + neg Cy5	2	1,53	3,4
GD3 PE + CLIPR Cy5	15,1	3,9	9,4

	FL-2	FL-3	FL-4	a	b	**FRET**
GD3 PE	13,3	2	-0,1	6,65		
tubulin Cy5	0	-0,8	20,09		-25,1125	
GD3 PE + tubulin Cy5	13,1	2,37	6			**0,26962**

**anti-Fas 60 min**	**FL-2**	**FL-3**	**FL-4**
neg PE	1,5	1,27	2,35
GD3 PE	14,2	3,7	2,31
neg Cy5	2,3	6,4	2,24
tubulin Cy5	1,06	4,74	21
neg PE + neg Cy5	1,21	1,63	3,8
GD3 PE + tubulin Cy5	15,36	3,19	20,81

	FL-2	FL-3	FL-4	a	b	**FRET**
GD3 PE	12,7	2,43	-0,04	5,226337		
tubulin Cy5	-1,24	-1,66	18,76		-11,3012	
GD3 PE + tubulin Cy5	14,15	1,56	17,01			**0,2293**

Figure S1
