# Supplementary material for: Role of GD3-CLIPR-59 Association in Lymphoblastoid T Cell Apoptosis Triggered by CD95/Fas
Source: PLoS One. 2010 Jan 5;5(1):e8567. doi: 10.1371/journal.pone.0008567 (PMC2797139; doi:10.1371/journal.pone.0008567)
Supplement: Figure S2 — Statistical analyses (ANOVA) of FRET data in Figure 4C. Data (A), tabular results (B) and narrative results (C) are included. (1.10 MB PDF) [file pone.0008567.s002.pdf]

	X Values	A						B					
	time	controls						clipr59					
	X	A:Y1	A:Y2	A:Y3	A:Y4	A:Y5	A:Y6	B:Y1	B:Y2	B:Y3	B:Y4	B:Y5	B:Y6
1	0	1.7	1.5	2.1	1.9	1.6	1.4	1.5	1.8	2.0	1.7	2.1	2.9
2	15	10.0	11.0	8.5	9.2	13.8	13.5	1.8	2.2	3.7	3.5	2.0	1.8
3	30	42.5	49.0	37.5	39.0	47.0	43.0	10.2	8.5	8.0	11.2	8.9	7.8
4	45	38.5	33.1	37.2	30.5	43.2	33.5	23.2	26.0	20.5	18.2	17.1	27.0
5	60	3.5	4.0	3.0	4.2	4.8	4.5	43.0	31.2	40.6	35.4	31.3	28.5
6	75	1.4	2.9	1.3	1.6	1.1	2.1	45.2	38.5	34.0	43.2	44.1	31.5
7	90	3.1	2.4	1.8	1.3	1.4	2.0	45.0	42.4	36.8	37.3	38.5	40.0
8	120	3.0	2.3	2.1	1.9	1.5	3.0	1.7	1.9	2.6	2.8	1.4	1.6

A

		A	B	C	D
	Parameter	Data Set-A	Data Set-B	Data Set-C	Data Set-D
		Y	Y	Y	Y
1	Table Analyzed	Data 3			
2					
3	Two-way RM ANOVA	Matching by cols			
4					
5	Source of Variation	% of total variation	P value		
6	Interaction	56.61	P<0.0001		
7	Time	37.03	P<0.0001		
8	siRNA	3.63	P<0.0001		
9	Subjects (matching)	0.4614	0.1893		
10					
11	Source of Variation	P value summary	Significant?		
12	Interaction	***	Yes		
13	Time	***	Yes		
14	siRNA	***	Yes		
15	Subjects (matching)	ns	No		
16					
17	Source of Variation	Df	Sum-of-squares	Mean square	F
18	Interaction	7	14780	2111	249.0
19	Time	7	9668	1381	162.9
20	siRNA	1	948.2	948.2	78.70
21	Subjects (matching)	10	120.5	12.05	1.421
22	Residual	70	593.5	8.478	
23					
24	Number of missing values	0			
25					
26	Bonferroni posttests				
27					
28	controls vs. clipr59				
29	siRNA	controls	clipr59	Difference	95% CI of diff.
30	0.0000	1.700	2.000	0.3000	-4.544 to 5.144
31	15.00	11.00	2.500	-8.500	-13.34 to -3.656
32	30.00	43.00	9.100	-33.90	-38.74 to -29.06
33	45.00	36.00	22.00	-14.00	-18.84 to -9.156
34	60.00	4.000	35.00	31.00	26.16 to 35.84
35	75.00	1.733	39.42	37.68	32.84 to 42.53
36	90.00	2.000	40.00	38.00	33.16 to 42.84
37	120.0	2.300	2.000	-0.3000	-5.144 to 4.544
38					
39	siRNA	Difference	t	P value	Summary
40	0.0000	0.3000	0.1739	P > 0.05	ns
41	15.00	-8.500	4.928	P<0.001	***
42	30.00	-33.90	19.65	P<0.001	***
43	45.00	-14.00	8.117	P<0.001	***
44	60.00	31.00	17.97	P<0.001	***
45	75.00	37.68	21.85	P<0.001	***
46	90.00	38.00	22.03	P<0.001	***
47	120.0	-0.3000	0.1739	P > 0.05	ns

B

Source of Variation	DF	Sum of Squares	Mean Square
Interaction	7.0	14780	2111
Time	7.0	9668	1381
siRNA	1.0	948.2	948.2
Subjects (matching)	10.0	120.5	12.05
Residual (Error)	70.0	593.5	8.478
Total	95.0	26110	

Does Time have the same effect at all values of siRNA?

Interaction accounts for 56.61% of the total variance.
F = 249.03. DFn=7 DFd=70
The P value is <0.0001
If there is no interaction overall, there is a less than 0.01% chance of randomly observing so much interaction in an experiment of this size. The interaction is considered extremely significant.

Since the interaction is statistically significant, the P values that follow for the row and column effects are difficult to interpret.

Does siRNA affect the result? (Are the curves different?)

siRNA accounts for 3.63% of the total variance (after adjusting for matching).
F = 78.70. DFn=1 DFd=70
The P value is <0.0001
If siRNA has no effect overall, there is a less than 0.01% chance of randomly observing an effect this big (or bigger) in an experiment of this size. The effect is considered extremely significant.

Does Time affect the result? (Are the curves horizontal?)

Time accounts for 37.03% of the total variance (after adjusting for matching).
F = 162.89. DFn=7 DFd=70
The P value is <0.0001
If Time has no effect overall, there is a less than 0.01% chance of randomly observing an effect this big (or bigger) in an experiment of this size. The effect is considered extremely significant.

Was the matching effective?

F = 1.42. DFn=10 DFd=70
The P value = 0.1893
If matching were not effective overall, there is a 19% chance of randomly observing an effect this big (or bigger) in an experiment of this size. The effect is considered not significant.

C

Figure S2
