# Supplementary figures and images for: Role of GD3-CLIPR-59 Association in Lymphoblastoid T Cell Apoptosis Triggered by CD95/Fas
Source: PLoS One. 2010 Jan 5;5(1):e8567. doi: 10.1371/journal.pone.0008567 (PMC2797139; doi:10.1371/journal.pone.0008567)

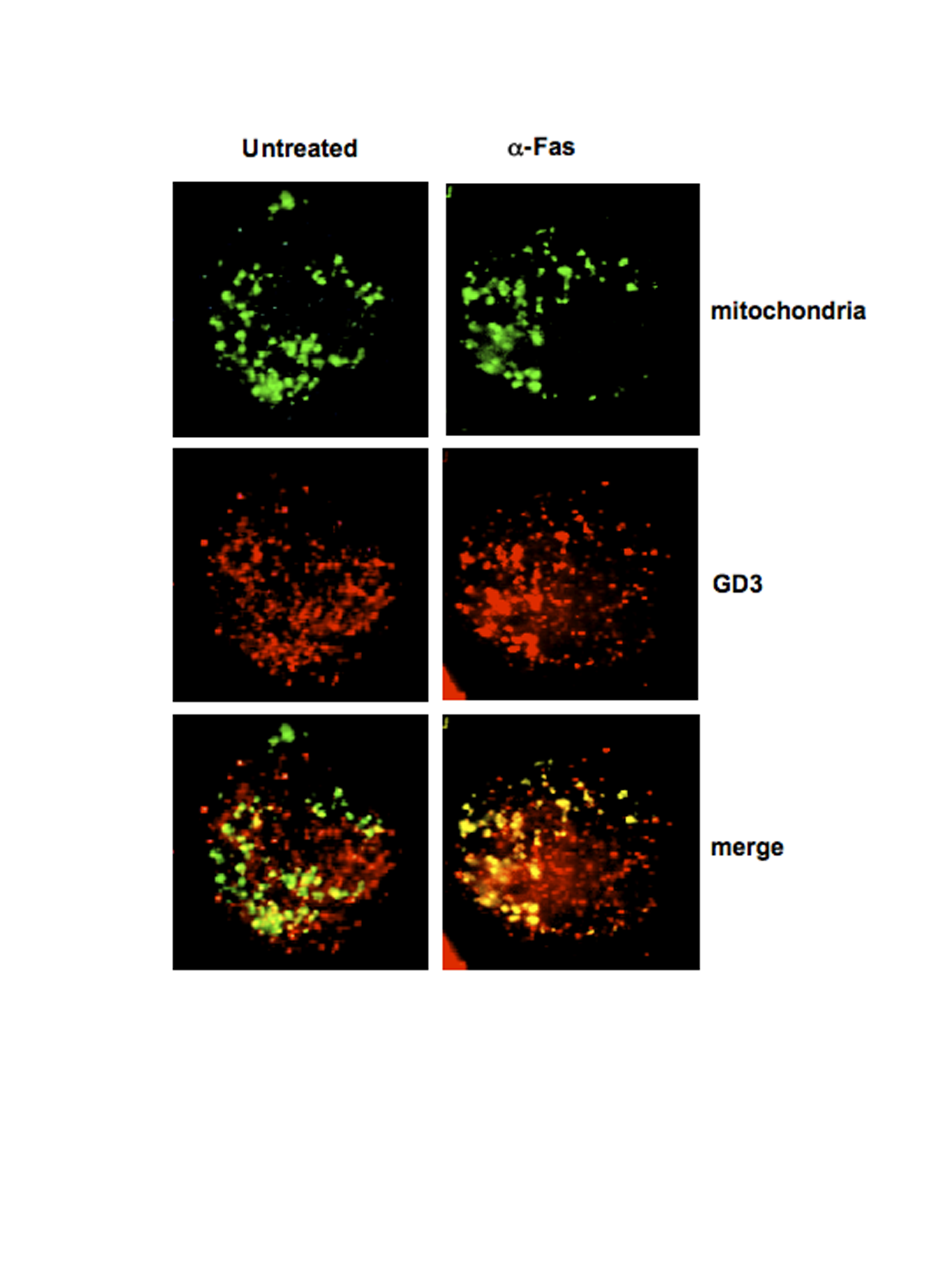

Supplement: Figure S3 — Immunofluorescence analysis of GD3/mitochondria association by R24 MoAb. Immunofluorescence analysis after double staining of GD3 (red) and mitochondria (green) in control and anti-CD95/Fas treated cells. Mitochondria were stained with MitoTracker-Green and GD3 with anti-GD3 R24 MoAb, followed by anti-mouse Alexa594. (18.08 MB TIF) [file pone.0008567.s003.tif]
